# Discrepancies in Mother–Child Reports of Child’s Exposure to Intimate Partner Violence: Associations With Externalizing Symptoms

**DOI:** 10.1177/08862605231173434

**Published:** 2023-05-25

**Authors:** Diogo Lamela, Inês Jongenelen

**Affiliations:** 1 Lusófona University, HEI-Lab, Porto, Portugal

**Keywords:** informant discrepancies, multiple informants, intimate partner violence, child exposure to intimate partner violence, externalizing symptoms

## Abstract

The type and frequency of children’s exposure to intimate partner violence (IPV) are considered as key variables in understanding children’s heightened risk of externalizing symptoms. Notably, children’s exposure to IPV has been primarily measured using mothers’ reports of their own victimization. However, mothers and children might differently perceive children’s exposure to physical IPV. To date, no research has investigated multi-rater reporting discrepancies in child’s exposure to physical IPV and whether such discrepancies would be linked to externalizing symptoms. This study aimed to identify patterns of mother–child discrepancies in child’s exposure to physical IPV and examine whether those patterns would be associated with children’s externalizing symptoms. Participants were mothers who have experienced police-reported male-perpetrated IPV and their children (*n* = 153; 4–10 years). Latent profile analysis identified three profiles of mother–child discrepancies: a concordant group reporting high IPV exposure; a discordant group with mothers and children reporting high and low child’s IPV exposure, respectively; a second discordant group with mothers and children reporting low and moderate IPV exposure, respectively. Profiles of mother–child discrepancies were differentially associated with children’s externalizing symptoms. Findings suggest that discrepancies among informants’ ratings of children’s IPV exposure might have important implications for measurement, assessment, and treatment.

Intimate partner violence (IPV) is a critical social issue that poses a significant threat to the human rights and well-being of women and their children (Sardinha et al., 2022). While the presence of ecological protective factors and coping skills may promote resilient responses in some children exposed to physical IPV, exposure to this type of violence remains as a significant risk factor for acute difficulties in psychological adjustment, including externalizing symptoms such as oppositional behavior, aggression, hyperactivity, violent peer and intimate relationships, and delinquency (Fong et al., 2019; Pinto et al., 2019; Yule et al., 2019). Several studies have also demonstrated that children’s exposure to physical IPV can hinder their progress in achieving developmental tasks, thereby increasing the risk of developing externalizing symptoms later in life (Carpenter & Stacks, 2009; Davies et al., 2015). For example, exposure to physical IPV has been linked to difficulties in emotion recognition, which can negatively affect a child’s ability to communicate and interact with others (Bender et al., 2022). Children who are exposed to physical IPV may also develop beliefs that violence is justifiable, which, combined with a greater perception of IPV-related threat, can lead to greater behavioral dysregulation and more aggressive and hostile behavior in interpersonal interactions (Jouriles et al., 2014). In addition to these direct effects, physical IPV can have indirect effects on externalizing symptoms by negatively impacting mothers’ mental health, emotional responsiveness, and parenting practices (Lamela et al., 2018; Yetter, 2022).

Despite the consistency of these findings, a growing body of literature suggests that the relationship between IPV exposure and children’s externalizing symptoms may not be linear and that it is important to account for individual differences that may explain this variability (Vu et al., 2016). The type of exposure is emerging as a key variable to explain interindividual variability in externalizing symptoms since children who directly witnessed or were involved in the violence exhibited a higher risk of externalizing symptoms (Latzman et al., 2017). Notably, the children’s exposure to IPV has not been directly measured in the past empirical research but instead inferred from the mother’s report of her own victimization or police/child protective service reports (Latzman et al., 2017; Wolfe et al., 2003). This is a critical methodological issue since this measurement option might not control significant factors that could contribute to an accurate assessment of the level of child’s exposure and the subsequent impact on child’s behavior. These factors include the timing of the stressor, the individual’s perception of threat, and informant bias. For example, prior theoretical and empirical research has proposed that accuracy in self-reporting may be influenced by depression symptoms, which can trigger a negative perceptual bias in the evaluation of a target construct (Madsen et al., 2020; Richters & Pellegrini, 1989). This bias can lead to the underreporting of events or experiences that are perceived as negative or distressing.

To overcome this methodological weakness, the current study applied a multi-informant approach to examine mothers’ and children’s reports of children’s exposure to IPV. This approach assumes that capturing and interpreting discrepancies between informants add meaningful information to clinical assessment as the level of informant discrepancies might reflect contextual variations in behavior or informants’ perspectives on behavior (i.e., how persons perceive, interpret, and recall the same events differently) (De Los Reyes, 2013). This assumption is advocated by the attribution bias context model, which posits that the level of discrepancy between informants in the assessment of child behavior reflects: (a) individual interpretations of child behaviors, (b) individual interpretation of behavior (e.g., whether a behavior is problematic/abnormal), and (c) the context in which information is collected (De Los Reyes & Kazdin, 2005).

Research has found that discrepancies between parent and child ratings of family functioning are linked with poor child adjustment and that greater discrepancies in parenting practices are associated with higher levels of externalizing symptoms (De Los Reyes et al., 2019; Hou et al., 2020; Nichols & Tanner-Smith, 2022). Additionally, a separate body of literature has proposed that children’s internal working models of attachment, which act as cognitive schemas that guide the processing of attachment-related social behavior, may play a role in explaining reporting discrepancies (Dykas & Cassidy, 2011). Research has shown that attachment security can significantly impact a child’s perception and reporting of their parents’ parenting behavior, as well as the level of discrepancy between parent and adolescent reports of conflict (Ehrlich et al., 2011; Penner et al., 2020). Furthermore, K. L. Goodman et al. (2010) suggest that discrepancies in parent–child reports of child victimization reflect lower quality parent–child relationships, which may impede children from disclosing victimization experiences to their caregivers. Such discrepancies can result in less effective responses to violence exposure, compromising children’s coping skills, and leading to subsequent psychological difficulties (K. L. Goodman et al., 2010).

Despite the empirical consistency and theoretical advancements on informant discrepancies regarding family functioning, no previous research, to our knowledge, has addressed whether or how parent–child discrepancies in child’s exposure to physical IPV would be associated with externalizing symptoms. Therefore, we first sought to identify patterns of discrepancies between mothers and children in their reports of child exposure to physical IPV. We then examined whether these patterns would be associated with the levels of children’s externalizing symptoms. We adopted a multi-informant approach to evaluate children’s externalizing symptoms, based on compelling empirical evidence about the contextual variability in their manifestation (Drabick et al., 2007; Genachowski et al., 2022;). Drawing from clinical- and theory-based recommendations (Dirks et al., 2012; Rescorla et al., 2019), we collected data from both mothers and teachers to explore the usefulness of patterns of discrepancies in mother–child reporting of children’s exposure to IPV in identifying potential variations in their behavior across different contexts. This multi-informant approach to assessing externalizing symptoms has been employed in previous research involving children exposed to IPV (Capaldi et al., 2020; Lamela et al., 2018; Zarling et al., 2013).

## Method

### Participants and Procedures

Participants consisted of 153 Portuguese mothers and their children (aged 4–10 years). Mothers had to have experienced police-reported male-perpetrated IPV during the last year. Participants were recruited from Child Protective Services and shelter residences from all regions of Portugal. To be enrolled in the research, mothers had to be at least 18 years old, the child had to live with the mother, either at home with the abusive partner or in the IPV shelter, and the child must not have a diagnosed cognitive or sensory disorder. Demographic data are summarized in Table 1.

**Table 1.** Sample Demographics, Profile Differences on Demographics, Child Exposure to IPV, Maternal Variables, and Child Externalizing Symptoms.

*M* (*SD*) or *n* (%)	Total Sample (*N* = 153)		Profiles		Statistic (Effect Size)	Group Contrasts
		P1: M++/C++ (*n* = 55)	P2: M++/C− (*n* = 35)	P3: M− /C+ (*n* = 63)		
Clustering variables						
Children’s exposure to IPV (mother) (VEX-R)	1.68 (0.89)	2.39 (0.53)	2.16 (0.61)	0.79 (0.33)	*F* = 181.61*** (*f*^2^ = 1.06)	P1, P2 > P3
Children’s exposure to IPV (child) (VEX-R)	1.60 (0.77)	2.24 (0.48)	0.79 (0.44)	1.48 (0.64)	*F* = 78.29*** (*f*^2^ = 0.76)	P1 > P3 > P2
Demographics						
Mothers’ age	36.26 (7.52)	35.67 (7.80)	35.00 (7.59)	37.79 (6.84)	*F* = 2.03 (*f*^2^ = 0.44)	ns
Mothers’ marital status (married/cohabitation)	90 (58.8%)	30 (54.5%)	19 (54.3%)	41 (65.1%)	χ^2^ = 1.73 (*V* = 0.11)	ns
Mothers’ dwelling status (shelter)	79 (51.6%)	33 (60.0%)	22 (62.9%)	24 (38.1%)	χ^2^ = 7.93** (*V* = 0.23)	P1, P2 > P3
Mothers’ education (<9th grade)	85 (53.5%)	34 (61.8%)	15 (42.9%)	32 (50.8%)	χ^2^ = 3.29 (*V* = 0.15)	ns
Physical IPV during pregnancy (yes)^b^	77 (51.3%)	39 (73.6%)	17 (48.6%)	21 (33.9%)	χ^2^ = 18.18 (*V* = 0.35)	P1 > P2, P3
Child’s age	7.25 (1.94)	7.56 (1.74)	7.26 (1.98)	7.10 (2.06)	*F* = 0.88 (*f*^2^ = 0.14)	ns
Child’s sex (boys)	85 (55.5%)	28 (50.9%)	17 (48.5%)	34 (53.9%)	χ^2^ = 0.28 (*V* = 0.04)	ns
Child’s diagnosed health problems (yes)	45 (29.4%)	12 (32.8%)	10 (28.6%)	23 (36.5%)	χ^2^ = 3.07 (*V* = 0.14)	ns
Mothers’ exposure to physical IPV (CTS2)	163.10 (108.99)	206.54 (91.33)	173.20 (115.72)	119.55 (104.20)	*F* = 10.77*** (*f*^2^ = 0.85)	P1, P2 > P3
Mothers’ depression symptoms (BSI)	11.54 (5.74)	12.46 (5.71)	11.26 (4.91)	10.90 (6.15)	*F* = 1.13 (*f*^2^ = 0.30)	ns
Children’s externalizing symptoms (mother) (SDQ)	9.80 (4.32)	10.75 (3.52)	10.74 (5.01)	8.46 (4.26)	*F* = 5.48** (*f*^2^ = 0.55)	P1, P2 > P3
Children’s externalizing symptoms (teacher) (SDQ)	11.07 (5.29)	13.42 (3.91)	11.36 (6.25)	9.04 (5.20)	*F* = 4.51** (*f*^2^ = 0.85)	P1 > P2, P3

P1: M++/C++ = Concordant on High Exposure to Physical IPV. P2: M++/C− = Discordant Reports between mother and child, with high mother, low child report. P3: M−/C+ = Discordant Reports between mother and child, with low mother, moderate child report. Significant group differences at *p* ≤ .05 (Tukey–Kramer post hoc tests). Child exposure to physical IPV (VEX-R) scores differed between informants for discrepant profiles (high mother/low child exposure to physical IPV profile: 1.37 points, *p* < .001, *d* = 2.58); low mother/moderate child exposure to physical IPV profile: 0.79 points, *p* > .001, *d* = 1.36), but not for the agreement profile (high exposure to physical IPV: 0. 15 points, ns). VEX-R = Violence Exposure Scale for Children—Revised; CTS2 = Revised Conflict Tactics Scales; BSI = Brief Symptom Inventory; SDQ = Strengths and Difficulties Questionnaire; IPV = intimate partner violence.

a *N* = 150.

** *p* < .01. ***p < .001.

Participants were recruited from 12 Child Protective Services and 117 domestic violence shelters. The professionals at Child Protective Services and shelter residences approached eligible participants and provided information about the research and ethical procedures. Women who expressed their interest in participating were invited to a meeting where the research aim and confidentiality procedures were explained in detail. Following the provision of written informed consent, trained female research assistants administered the research protocol at Child Protective Services or shelter facilities. Women who participated in the study received compensation in the form of a voucher from a local department store. All mothers gave permission for their child’s early childhood educator or primary teacher to be contacted to complete an assessment protocol about the child’s adjustment. Mothers were informed that the teachers’ participation aimed to collect information about the child’s behavior in the school setting, and that no personal information regarding IPV referral would be shared with the teachers. The research team obtained teachers’ contact information from the mothers and contacted them by telephone and invited them to participate. To protect the privacy and safety of mothers and children, a broad research objective (“understand the effect of family relationships on children’s psychological adjustment”) was provided to the teachers. The assessment protocol and consent form were sent to the teachers via email and one reminder email was sent after a month. The response rate was 38.1% and no compensation was offered to the teachers. No significant differences were found between participating and nonparticipating teachers in children’s age; *t* = −0.853 ns, and in children’s gender; χ^2^(1) = 1.94 ns.

The research procedures underwent ethical review and were approved by both the institutional review board and the Portuguese Data Protection Authority. To ensure compliance with the World Health Organization’s ethical and safety recommendations for research on violence against women, we implemented measures such as obtaining informed consent, safeguarding confidentiality and privacy, and providing appropriate support and resources for participants who may experience distress due to the study. In cases where participants were living with abusive partners, we collected data in CPS facilities during prescheduled appointments made by professionals to guarantee a safe space. We followed safety protocols in child protective services facilities and shelters during data collection to ensure participants’ safety and privacy. To ensure the safety and rights of participants, the research assistants underwent trauma-informed training provided by researchers with substantial clinical experience in working with IPV-exposed women and children. The training included an initial informational module on IPV, a second module on the measures of the research protocol, and a final practice module consisting of rotary role-play and role model sessions to administer the research protocols.

### Measures

#### Exposure to Physical IPV

Mothers’ and children’s reports of children’s exposure to physical IPV were assessed using the witnessing subscale of the Violence Exposure Scale for Children—Revised (VEX-R) (Fox & Leavitt, 1996). The VEX-R comprises child and parent versions with similar items. The child’s version adopts a cartoon format, using cards illustrating violent acts. Children and parents are asked to indicate the frequency of the child’s exposure to those acts as a witness. The frequency of exposure is rated on a four-point scale (from 0 “*never*” to 3 “*lots of times*”). The witnessing subscale of the VEX-R comprises 21 items assessing the child’s exposure to different types of threatening or criminal behaviors (e.g., the child witnesses a caregiver stealing or being arrested or hitting another caregiver). For the current analyses, we only included the 16 items that specifically assessed exposure to physical violence. Trained examiners administered the VEX-R to children face-to-face. The Portuguese version of the VEX-R was validated for children in ages between 4 and 10 years and revealed adequate psychometric properties (Sousa, 2015). The Cronbach’s alpha reliability in the current sample was .80.

#### Physical IPV

Mothers’ exposure to physical IPV was measured by the Revised Conflict Tactics Scales (CTS2) (Straus et al., 1996). The CTS2 is scored by summing the frequency of each of the behaviors for each scale, with higher scores indicating more frequent aggression. Responses were made on a six-point Likert-type scale ranging from never to more than 20 times. A composite score of mothers’ exposure to physically abusive behaviors in the last 12 months (i.e., IPV chronicity) was computed by summing the frequency of items of the physical assault and injury scales. The 12-item physical assault scale measures the presence and frequency of exposure to certain violent behaviors perpetrated by an intimate partner (e.g., beaten up by a partner). The six-item injury subscale assesses how often certain types of injury occurred as a result of physical assault by an intimate partner (e.g., the participant was cut or bleeding). The Portuguese version of the CTS2 demonstrated good psychometric properties (Paiva & Figueiredo, 2006). For the current sample, coefficient alpha for the physical IPV composite was .86.

#### Maternal Depression Symptoms

Maternal depression symptoms were assessed using the depression scale of the Brief Symptom Inventory (BSI) (Derogatis & Melisaratos, 1983). This six-item scale measures individuals’ depressed mood, sadness, loss of interest in life activities, unworthiness/worthlessness, hopelessness, loneliness, and vulnerability to criticism. Participants reported their levels of distress over the previous 2 weeks, using a five-point Likert scale (from 0 “*not at all*” to 4 “*extremely*”). Higher scores reflect greater levels of depression symptoms. A consistent body of clinical empirical research suggested the reliability and validity of the BSI depression for the screening of depression symptoms (Callender et al., 2012; Flens et al., 2022). The Portuguese version of the depression scale of the BSI revealed good internal consistency (Canavarro, 1999). In the current sample, Cronbach’s alpha was .82.

#### Children’s Externalizing Symptoms

The Strengths and Difficulties Questionnaire—Parent and Teacher Forms (SDQ) (R. Goodman, 1997) was administered to mothers and teachers to assess children’s externalizing symptoms. Externalizing symptoms were measured by computing a composite score obtained from the sum of the scores of two SDQ subscales: hyperactivity/inattention and conduct problems ( A.Goodman et al., 2010). Items are rated on a three-point response scale (from 0 “not true” to 2 “certainly true”), with each subscale constructed as the sum of five items. Higher scores reflect greater externalizing symptoms. The Portuguese version of the SDQ revealed good psychometric properties (Ferreira et al., 2021). The Cronbach’s alpha reliabilities for the Externalizing Symptoms composite were .72 (mothers form) and .75 (teacher form).

### Data Analysis Strategy

We conducted the latent profile analyses (LPA) to investigate patterns of discrepancies in mother–child reports of child’s exposure to physical IPV. To inspect the best solution, we estimated models with one, two, three, four, and five latent profiles (models constraining variances across classes to be equal and forcing covariances to 0). The optimal solution selection was determined based on the best-fitting values on five fit indices (Table 2). Subsequently, one-way analyses of variance and chi-square tests were conducted to examine differences between the mother–child discrepancy profiles in sociodemographics, family variables, mothers’ depression symptoms, and children’s externalizing symptoms (mother and teacher reports). Finally, Tukey–Kramer post hoc tests were conducted to identify significant differences between groups. LPA were conducted using the tidyLPA package in R (Rosenberg et al., 2018).

**Table 2.** Fit Statistics for the Latent Profile Models.

Classes	AIC	BIC	SABIC	Entropy	BLRT (*p*-value)	*n*_min	*n*_max
1	788.32	800.60	788	1	—	—	—
2	759.42	785.90	759	0.771	34.9 (0.009)	0.421	0.579
3	743.78	775.47	744	0.826	18.6 (0.009)	0.283	0.428
4	764.53	804.43	763	0.658	−11.8 (0.683)	0.199	0.409
5	770.18	819.28	769	0.645	0.350 (0.723)	0.101	0.403

Entropy, the certainty of classification across cases; BLRT comparing model to model (*k* − 1), where *k*, number of classes; significance of BLRT indicates improved model fit; *n*_min, the proportion of the sample assigned to the smallest class; *n*_max, the proportion of the sample assigned to the largest class. AIC = Aikake Information Criterion; BIC = Bayesian Information Criterion; SABIC = Sample-Adjusted Bayesian Information Criterion; BLRT = bootstrapped likelihood test.

## Results

We examined fit statistics for the five models estimated in the LPA. The model with three classes revealed optimal fit (Table 2). Next, we characterized the three profiles based on the most salient dimensions in mother and child reports on child exposure to physical IPV (Table 1). In Profile 1, both mothers and children rated as children being highly exposed to physical IPV, referred to here as the high exposure to physical IPV (Agreement). In Profile 2, mothers rated their children as highly exposed to physical IPV. In contrast, children self-reported low exposure to physical IPV. Profile 2 referred to here as the high mother/low child exposure to physical IPV profile (discrepant). Finally, mothers in Profile 3 reported low child exposure to physical IPV. In contrast, children rated moderate exposure to physical IPV, referred to here as the low mother/moderate child exposure to physical IPV profile (discrepant).

Mothers in the high exposure to physical IPV and the high mother/low child exposure to physical IPV profiles were more frequently exposed to physical violence than mothers in the low mother/moderate child exposure to physical IPV profile. A higher proportion of mothers in the earlier two profiles were living in IPV shelters. Profiles also differed in IPV exposure during pregnancy. We then tested whether profiles would differ in terms of maternal depression symptoms. No significant differences were found in mothers’ depression symptoms across the three profiles (all *p*s > .05).

Profiles differed in terms of child externalizing symptoms (Table 1). In particular, the high exposure to physical IPV and the high mother/low child exposure to physical IPV profiles exhibited significantly higher externalizing symptoms as reported by mothers compared with the low mother/moderate child exposure to physical IPV profile. In addition, the High Exposure to Physical IPV reported the highest levels of externalizing symptoms as reported by teachers. No differences between the discrepant profiles were found in externalizing symptoms as reported by teachers.

## Discussion

To our knowledge, this was the first study to apply a person-centered approach to capture profiles of informant discrepancies in child’s exposure to IPV and how such patterns were associated with child’s externalizing symptoms. Our results showed that the three profiles of mother–child discrepancies in child’s exposure to IPV were differently associated with externalizing symptoms as reported by mothers and teachers. In particular, we found that mothers’ reports of externalizing symptoms did not differ among the two profiles, with mothers rating high child’s exposure to IPV, regardless of children’s report (Profiles 1 and 2). On the other hand, teachers reported higher levels of externalizing symptoms in children of the agreement profile than in children of the two mother–child discrepancy profiles. These results suggest that informant discrepancies may reflect variations between mothers and children in how they experience IPV episodes and variations in children’s expression of externalizing behaviors across different settings (home vs. school) (De Los Reyes, 2013). In particular, mothers who reported being victims of more frequent violent behaviors (Profiles 1 and 2) also reported children’s higher exposure to IPV and higher externalizing symptoms. These two profiles also exhibited a higher proportion of dyads living in shelters. Taken together, these findings suggest that mothers and children of these two profiles were exposed to higher adversity and lower protection factors, as mothers scored higher on children’s exposure to IPV were more likely to seek shelters, have also reported experiencing more frequent episodes of IPV, and their children have more externalizing symptoms.

Surprisingly, we also found that children’s and mothers’ reports of child exposure were more similar at higher levels of intimate violence. The high agreement in higher child’s exposure to IPV is unexpected, as prior research suggested that parents underestimate the degree to which their conﬂicts are witnessed by their children (Holt et al., 2021; van Rooij et al., 2015). Similarly, children of the agreement and discrepant profiles (Profiles 1 and 2, respectively) exhibited similar levels of externalizing symptoms as reported by mothers. These results are only partly consistent with prior theoretical developments that proposed that higher mother–child agreement in child victimization is related to lower adjustment problems in children (K. L. Goodman et al., 2010). However, the compelling finding that mothers of Profile 1 (agreement on high exposure to IPV) reported more physical abuse during pregnancy than the other two profiles may suggest that these dyads may be victims of a more severe and chronic dynamics of IPV with detrimental impact children’s behavioral regulation, as suggested earlier (Silva et al., 2018).

Unexpectedly, the study did not find any differences in depression symptoms among the identified profiles. This finding suggests that maternal depression symptoms did not play a significant role in the level of agreement between informants. Although previous research has documented that depression symptoms can interfere with mothers’ ability to evaluate whether the child would interpret violent interactions as abusive behaviors and recall stressful episodes (Aisenberg et al., 2007; Lamela et al., 2017; Pointet Perizzolo et al., 2022), our study did not find empirical evidence of a similar pattern of findings. Other factors, such as the severity and frequency of IPV and children’s appraisals of IPV, may have had a more significant impact on informant discrepancies than maternal depression symptoms. Further research should explore psychological and social factors to fully understand the relationship between informant-reported discrepancies in children’s exposure and IPV and externalizing symptoms.

Taken together, our findings suggest that using a person-centered approach to identify profiles of discrepancies in children’s exposure to IPV can provide meaningful insights into the dynamics of IPV and children’s externalizing symptoms that cannot be captured by relying solely on one informant. For instance, our study found that children from Profiles 1 and 2 had similar levels of externalizing symptoms as reported by mothers, despite their significant differences in IPV exposure according to children’s self-reports. This finding is consistent with previous research that has shown a stronger association between externalizing symptoms and mothers’ reports of their children’s exposure to IPV, rather than children’s own reports (Harding et al., 2013). However, it highlights the potential for varying conclusions regarding the correlates of externalizing symptoms when relying solely on one informant rather than incorporating information from multiple informants.

The current study presents some limitations. First, due to the study’s cross-sectional nature, it is unclear whether discrepancy profiles predicted later externalizing symptoms or whether the relationship between mother–child discrepancies and child adjustment problems is bidirectional. Second, we only included mothers who reported IPV to the police. As the IPV dynamics and severity may differ between reported and unreported cases, our results may not be generalizable to women who did not report IPV to the police. It is also important to recognize that our results may not necessarily generalize to other cultures, as cultural factors may play a significant role in shaping the experience and reporting of IPV (Satyen et al., 2019). Additionally, research is called for to explore how other forms of diversity, such as socioeconomic status and children’s developmental stage, may intersect with IPV and impact the reporting discrepancies of violence. Third, mothers’ IPV victimization was measured by the frequency of exposure to physically abusive behaviors acts. Future research should discriminate the severity and type of violent acts, as the threat level might impact children’s assessment of the situational cues of a specific marital interaction. Future research should also investigate the sources of the discrepancy in child victimization, focusing on cases where children reported lower exposure to IPV than their mothers. In addition, our results might be extended by examining how informant agreement on children’s strategies to regulate their exposure to interparental conflict (e.g., avoidance of conflict interference) would be differentially associated with children’s psychological adjustment, as suggested recently (Holt et al., 2021). Future studies should also examine whether patterns of informant discrepancy in exposure to physical IPV would be replicated in exposure to other forms of IPV.

Our results expand previous literature by using a multi-informant approach to measure children’s exposure to physical IPV. In addition, both agreement and high disagreement about high children’s exposure to IPV were linked to higher externalizing symptoms, suggesting that the frequency of maternal exposure to physical IPV may be associated with patterns of response between mother–child dyads in high-risk populations. Analyzing informant-reported discrepancies can provide several advantages in research and clinical settings. Firstly, examining discrepancies between different informants can highlight potential areas of concern and disagreement, which can improve the assessment’s accuracy and the intervention’s efficacy. Moreover, informant discrepancies can provide valuable information about the children’s social context and the nature of the informant’s relationship with them. This information can be utilized to design interventions to the children’s and mothers’ specific needs, as they might recall, perceive, and interpret IPV exposure differently. Our study highlights the importance of developing interventions that address the mother–child dyad’s unique experiences and circumstances in the context of IPV. Specifically, the significant variability in how children and mothers perceive exposure to IPV, as reflected in different reporting discrepancies profiles, emphasizes the need for tailored interventions that identify and leverage individual and dyadic strengths, such as coping skills and support networks, to empower them to overcome IPV-related adversity and promote healing and growth. This approach acknowledges the unique experiences and circumstances of the mother–child dyad rather than focusing solely on the child or the mother and can lead to more effective and sustainable outcomes.
